# Brief Report: Exercise and Anxiety in Adults with Arthritis and Other Rheumatic Diseases: Support for Evidential Value

**DOI:** 10.1155/2018/2984671

**Published:** 2018-10-18

**Authors:** George A. Kelley, Kristi S. Kelley, Leigh F. Callahan

**Affiliations:** ^1^DA, FACSM, School of Public Health, Department of Biostatistics, West Virginia University, Morgantown, WV 26506-9190, USA; ^2^M.Ed., Research Instructor, School of Public Health, Department of Biostatistics, Robert C. Byrd Health Sciences Center, West Virginia University, PO Box 9190, Morgantown, WV 26506-9190, USA; ^3^PhD, Mary Link Briggs Distinguished Professor of Medicine, Professor, Departments of Social Medicine and Orthopaedics, Adjunct Professor, Department of Epidemiology, 3300 Thurston Bldg, Campus Box 7280, University of North Carolina, Chapel Hill, NC 27599-7280, USA

## Abstract

**Objective:**

Given the high prevalence of anxiety in adults with arthritis and other rheumatic diseases (AORD) and the subsequent need for interventions to reduce anxiety, this brief report sought to determine if evidential value exists to support the role of exercise for reducing anxiety in adults with AORD.

**Methods:**

Utilizing data from a prior meta-analysis, a recently developed approach,* P*-curve, was used to determine evidential value by assessing for publication bias and* p*-hacking. Binomial tests as well as the more robust Stouffer's test were used to examine for evidential value. To examine the influence of selected studies on* p-*curve results, findings were also examined by dropping the highest and lowest* p* values from the analysis.

**Results:**

The binomial test for evidential value was not statistically significant (*p* = 0.11) while the more robust Stouffer's test satisfied both conditions for evidential value (*p* = 0.002). Power analyses suggested a good fit for the observed* p*-curve. Results were generally robust when the least and most extreme values were excluded.

**Conclusions:**

The results of this study provide evidential support for the benefits of exercise on anxiety in adults with AORD.

## 1. Introduction

Arthritis and other rheumatic diseases (AORD) are a major public health problem among adults worldwide [1]. For example, in the United States (US) the prevalence of self-reported, doctor-diagnosed arthritis was estimated at 52.5 million (22.7%) adults and was projected to increase to 78.4 million (25.9%) by 2040 [2]. In 2013, arthritis-attributable medical costs as well as lost earnings in US adults with AORD were estimated to be $303.5 billion [3]. A major condition associated with AORD in adults is excessively high levels of anxiety, with recent research in the US reporting the prevalence to be approximately twice as high as depression (30.5% versus 17.5%) [4]. One potential therapy for decreasing anxiety is exercise. To support such, a recent systematic review with meta-analysis reported an overall standardized mean difference effect size reduction of -0.40 (95% CI, -0.65, -0.15) in anxiety as a result of exercise (aerobic, strength training or both) [5]. However, the vast majority of studies included (92.9%) were derived from peer-reviewed journals. This is potentially problematic given that researchers are inclined to report studies (publication bias) or analyses (*p*-hacking) that yield statistically significant results [6–10]. If present, this leads to results that do not accurately represent the truth with respect to the effects of exercise on anxiety in adults with AORD [11, 12]. While various methods to address these potential biases exist [13] and the prior meta-analysis found evidence of potential publication bias using the approach of Egger et al.[14], a recently developed and novel approach called* P*-curve has been developed for determining whether the observed effects in a study are true or, alternatively, whether they represent selective reporting and/or publishing of findings [8–10]. Thus, given the potential and important benefits of exercise for reducing anxiety in adults with AORD as well as the need to determine whether results suffer from the selective reporting and analyses of findings, the purpose of this brief report was to use the robust* P*-curve analysis approach [8–10] to confirm or refute the potential selective reporting and analyses of findings from this prior meta-analysis [5].

## 2. Materials and Methods

### 2.1. Data Source

Data for the current study were derived from a previous systematic review with meta-analysis of randomized controlled trials on the effects of exercise (aerobic, strength training, or both) in adults (mean ± standard deviation age = 54.2 ± 9.3 years) with AORD (osteoarthritis, rheumatoid arthritis, fibromyalgia), details of which have been described elsewhere [5]. Briefly, 14 studies that included 926 participants (539 exercise, 387 control) met all eligibility criteria. Length of training averaged 15.8 ± 6.7 weeks, frequency 3.3 ± 1.3 times per week and duration 28.8 ± 14.3 minutes per session. Overall, statistically significant exercise minus control reductions in anxiety were found (g = -0.40, 95% CI, -0.65, -0.15, tau^2^ = 0.14; Q = 40.3,* p* = 0.0004;* I*^2^ = 62.8%) [5]. To the best of our knowledge, this was the first systematic review with meta-analysis to ever examine the effects of exercise (aerobic, strength training, or both) on anxiety as a primary outcome in adults with AORD [5].

### 2.2. *P*-Curve Analysis

#### 2.2.1. Overview

*P*-curve analysis, described in detail elsewhere, is used to determine whether publication bias or* p*-hacking is present [8–10]. Simply stated, this novel test of evidential value corrects for publication bias using only statistically significant results in which half the* p*-curve has a right-skew test p<.05 or both the half and full* p*-curves have a right-skew test* p*<.01 [8–10]. It consists of a distribution of statistically significant probability (*p) *values limited only to those results in which* p* values are <0.05 [8–10]. It is based on the belief that true effects will result in right-skewed* p-*curves that include more low versus high statistically significant* p* values, i.e., more 0.01's versus 0.04's [8–10]. Thus,* p*-curves skewed to the right are considered to represent evidential value of true effects [8–10]. In accordance with the approach, only statistically significant results (all* p's *< 0.05) are entered for analysis. For the current study, and to be consistent with the conduct of the original meta-analysis [5], all data imputed for analysis consisted of* z-*scores with* p *values <0.05. The one study included in the original meta-analysis that was not published in a peer-reviewed journal, a dissertation, was excluded from the current analysis and also did not meet the required* p* <0.05 threshold for inclusion [15].

#### 2.2.2. Binomial Test

To determine if evidential value exists for exercise-induced changes in anxiety among adults with AORD, a binomial test with* p* values categorized as either low (*p* <0.025) or high (*p* > 0.025 up to <0.05) was conducted. A* p* value ≤ 0.05 was considered to represent evidential value, i.e., right skewness.

#### 2.2.3. Stouffer's Test

Because the binomial test does not distinguish between the different* p* values within the high and low categories, Stouffer's method was used to combine results across studies [10]. The rationale for using Stouffer's method was predicated on the fact that it is a more robust test and is less sensitive to outliers [16]. This more robust analysis combines half and full* p*-curves to draw inferences with respect to evidential value [10]. Half* p*-curve results that are right-skewed with a* p* value <0.05 or results in which both the half and full tests are right skewed with a* p* value <0.10 are considered to provide evidential value of a true effect of exercise on anxiety in adults with AORD [10].

#### 2.2.4. Binomial and Continuous Tests

Binomial and continuous tests were used to determine if evidential value was inadequate or absent when a 33% power test was <0.05 for the full* p*-curve or the half* p*-curve and binomial test were <0.1 [10]. A 33% power test was used based on previous recommendations that studies with such extremely low power will fail approximately 2 out of 3 times [9].

#### 2.2.5. Statistical Power across a Range of Values

Statistical power was also calculated by comparing the expected* p*-curve for each possible value ranging from 5% to 99% and then choosing the power level that results in an expected* p*-curve most similar to the actual* p*-curve [10]. Ninety-percent versus 95% confidence intervals were calculated so as to make it consistent with the one-sided test against a power null of 33% [10].

#### 2.2.6. Sensitivity of Results

To examine the influence of selected studies on* p-*curve results, findings were also examined by dropping the highest and lowest* p* values from the analysis [10]. All analyses were conducted using* P*-curve, version 4.052 [17].

## 3. Results

**Figure 1:**
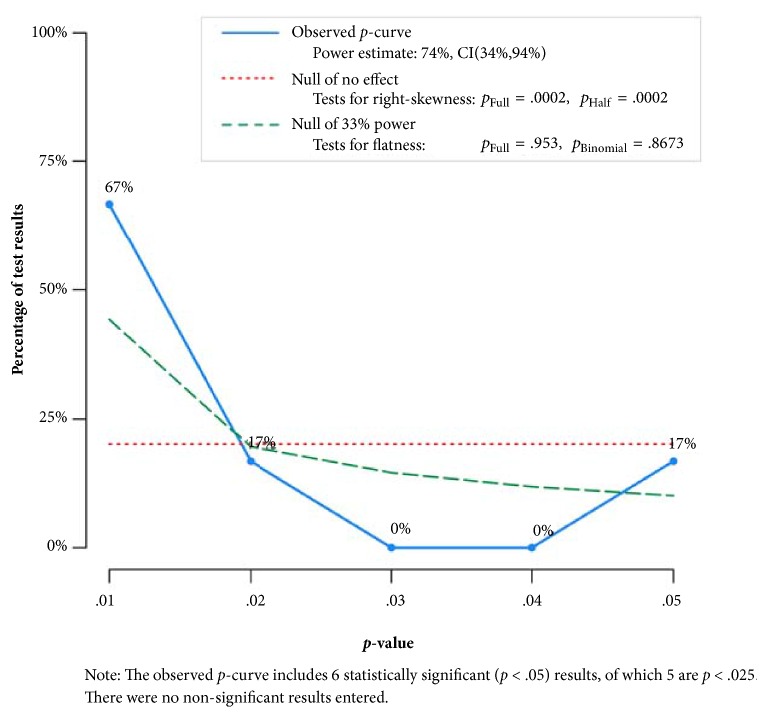
*P*-curve analysis results for changes in anxiety as a result of exercise in adults with AORD. The horizontal dotted red line represents what the distribution of* p *values would be under the assumption of no effect. The green dashed line suggests that sufficient power exists to detect evidential value under the null of 33% power. The blue line suggests that the data do not suffer from* p*-hacking because there is a predominance of* p* values (74%) that are 0.02 or smaller.

*P*-curve analysis results for changes in anxiety as a result of exercise in adults with AORD are shown in Table 1 and Figure 1 while study characteristics and calculations for each test entered into* p*-curve are shown in Supplementary files 1 and 2. Six* p *values ≤ 0.05 were included (Supplementary file 2). This represented 37.5% of the 16 results reported in the original manuscript [5]. The binomial test was not statistically significant (*p* = 0.11) while Stouffer's test satisfied both conditions for evidential value (*p* = 0.002 for both full and half* p*-curves). Similarly, binomial (*p* = 0.867) and full* p*-curve (p = 0.953) results were not suggestive that evidential value was inadequate or absent. Power analysis data are shown in Table 1, Figure 1 and Supplementary file 3. The V shape shown in Supplementary file 3 suggests a good fit for the observed* p*-curve. These findings indicate that if all studies were truly powered to 74%, half the time we would see a flatter* p*-curve than the one observed and half the time we would see a more right skewed one. Finally, results were generally robust when the most extreme values were either included or excluded (Supplementary file 4).

## 4. Discussion

The results of the current* P*-curve analysis suggest that publication bias and* p*-hacking does not exist and therefore, provides evidential support from the previously reported and positive findings regarding improvements in anxiety as a result of exercise in adults with AORD [5]. These findings are both biologically and psychologically plausible. Importantly, the current findings suggest that publication bias was not a potential source of statistical heterogeneity in the original meta-analysis, thereby strengthening our previous findings [5]. These results are in contrast to our previous findings [5] using the approach of Egger et al. [14]. Given the prevalence of anxiety in adults with AORD [4] as well as the need to identify the true effects of an intervention on an outcome, these findings are important from both a clinical and public health perspective. This is especially true since the previous meta-analysis found that the number-needed-to treat was 6 with a percentile improvement of 15.5% and an estimated 5.3 million inactive US adults with AORD improving their anxiety if they started exercising regularly (aerobic, strength training, or both) [5]. Importantly, the previously reported magnitude of effect (standardized mean difference = -0.40) is comparable to or greater than the use of anxiolytics for reducing anxiety [18]. Furthermore, anxiolytics are traditionally intended to target only anxiety and have potentially serious adverse events, including an increased risk for all-cause mortality [19]. In contrast, exercise is a relatively safe, low-cost intervention that results in a number of other physiological and psychological benefits beyond reductions in anxiety [20, 21]. While the results of this study are encouraging, it should be noted that one of the potential limitations of the* P*-curve approach is that it will often fail to detect studies that lack evidential value because such findings are only mildly left-skewed when a finding is considered to be* p*-hacked [9].

## 5. Conclusion

*P*-curve results provide evidential value in support of exercise for reducing anxiety in adults with AORD. These findings support our previous consensus using the Grading of Recommendations Assessment, Development and Evaluation (GRADE) Instrument [22] in that the quality of evidence was considered to be high and that further original studies on this topic would be very unlikely to change one's confidence in the overall estimate of effect [5].

## Figures and Tables

**Table 1:** *P*-curve results.

	Binomial Test	Continuous Test	
	(results with *p* <0.025)	(Aggregate with Stouffer Method)	
		Full *p*-curve (*p's* <0.05)	Half *p*-curve (*p*'s <0.025)

Studies contain evidential value (right skew)	*p* =0.1094	Z = 3.56 (*p*=0.0002)*∗*	Z= 3.59 (*p*=0.0002)*∗*
Studies evidential value, if any, is inadequate (flatter than 33% power)	*p* = 0.8673	Z=1.67 (*p* =0.953)	

Notes: *∗*, statistically significant, *p* ≤ 0.05; the nonsignificant binomial test for right skew suggests a lack of evidential value to dismiss *p*-hacking while the statistically significant results based on the more robust Stouffer's test (full and half *p*-curves) provide evidential value that *p*-hacking was not present. The nonsignificant findings for both binomial and full *p*-curve results for inadequacy (flatter than 33% power) suggest that sufficient power exists to detect evidential value.

## Data Availability

The data used to support the findings of this study are available from the corresponding author upon request.

## Supplementary Materials

Supplementary Materials Supplementary file 1: study characteristics table. Supplementary file 2: calculations for each test entered into* p*-curve. Supplementary file 3: diagnostic plot for power estimation. Supplementary file 4: plots for highest and lowest* p* values.
