# Supplementary material for: Brief Report: Exercise and Anxiety in Adults with Arthritis and Other Rheumatic Diseases: Support for Evidential Value
Source: Biomed Res Int. 2018 Oct 18;2018:2984671. doi: 10.1155/2018/2984671 (PMC6211156; doi:10.1155/2018/2984671)
Supplement: Supplementary Materials — Supplementary file 1: study characteristics table. Supplementary file 2: calculations for each test entered into p-curve. Supplementary file 3: diagnostic plot for power estimation. Supplementary file 4: plots for highest and lowest p values. [file 2984671.f1.docx]

Suppementary file 1. Study characteristics table.*

| **Study*** | **Study Design** | **Statistical Approach** | **Results**  **SMD + SE** | **Results Entered Into *P*-Curve (*Z*)** |
| --- | --- | --- | --- | --- |
| Minor et al., 1989 (pool) | RCT | Exercise minus control group changes using Hedge’s SMD effect size | 1.47 + 0.34 | 4.31 |
| Minor et al., 1989 (walk) | RCT | Exercise minus control group changes using Hedge’s SMD effect size | 1.27 + 0.36 | 3.58 |
| Tomas-Carus et al., 2007 | RCT | Exercise minus control group changes using Hedge’s SMD effect size | 1.12 + 0.37 | 3.05 |
| Gowans et al., 2001 | RCT | Exercise minus control group changes using Hedge’s SMD effect size | 1.01 + 0.35 | 2.90 |
| Tomas-Carus et al., 2008 | RCT | Exercise minus control group changes using Hedge’s SMD effect size | 1.00 + 0.39 | 2.57 |
| Schachter et al., 2003 | RCT | Exercise minus control group changes using Hedge’s SMD effect size | 0.61 + 0.30 | 2.02 |

Notes: *, modified *P*-curve discloure table, derived from Simonsohn U, Simmons JP, Nelson LD. Better *P*-curves: Making *P*-curve analysis more robust to errors, fraud, and ambitious *P*-hacking, a Reply to Ulrich and Miller (2015). *J Exp Psychol Gen* 2015;**144**: 1146-1152; RCT, randomized controlled trials; SMD + SE, standardized mean difference + standard error; References: Minor MA, Hewett JE, Webel RR, et al. Efficacy of physical conditioning exercise in patients with rheumatoid arthritis and osteoarthritis. Arthritis Rheum 1989;32(11):1396-405; Tomas-Carus P, Gusi N, Leal A*, et al.* [The fibromyalgia treatment with physical exercise in warm water reduces the impact of the disease on female patients' physical and mental health]. *Rheumatol Clin* 2007;3(1):33-37; Gowans SE, deHueck A, Voss S, et al. Effect of a randomized, controlled trial of exercise on mood and physical function in individuals with fibromyalgia. Arthritis Rheum 2001;45(6):519-29; Tomas-Carus P, Gusi N, Hakkinen A, et al. Eight months of physical training in warm water improves physical and mental health in women with fibromyalgia: a randomized controlled trial. J Rehabil Med 2008;40(4):248-52; Schachter CL, Busch AJ, Peloso PM, et al. Effects of short versus long bouts of aerobic exercise in sedentary women with fibromyalgia: a randomized controlled trial. Phys Ther 2003;83(4):340-58.

Supplementary file 2. Calculations for each test entered into *p*-curve.


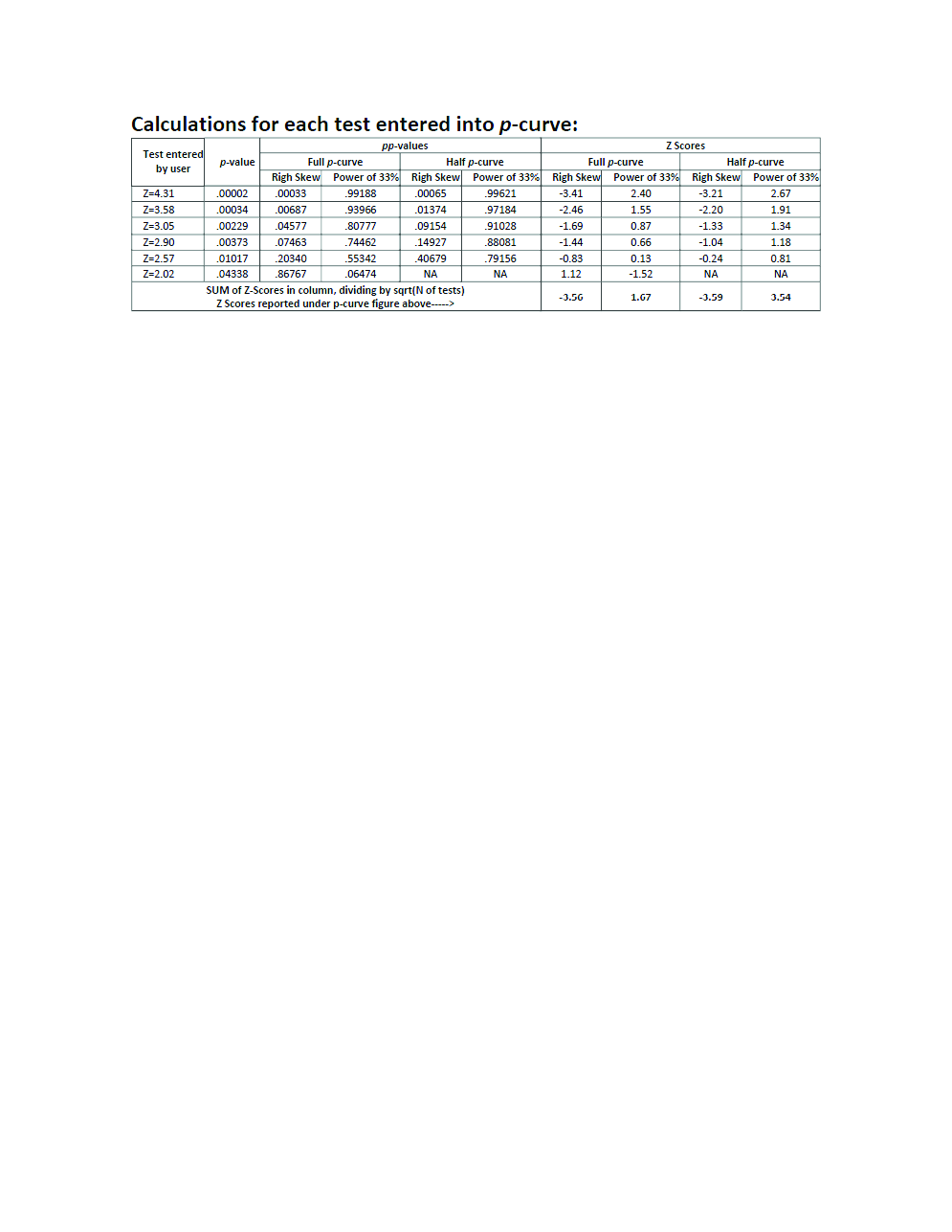


Notes: The “Test entered by user” column represents the *Z*-value from each of the six included studies while the *p*-value column represents the corresponding *p*-value for each *Z*-value. Smaller *p-*values suggest a lack of *p*-hacking. The *pp*-values represent the probability of the *p*-values from each included study, tested for right skewness, as well as the power to detect for evidential value using both full and half *p*-curve tests. The Z-scores column represents the same general approach as the *pp*-values column but with the use of Z versus *pp*-values.

Supplementary file 3. Diagnostic plot for power estimation.


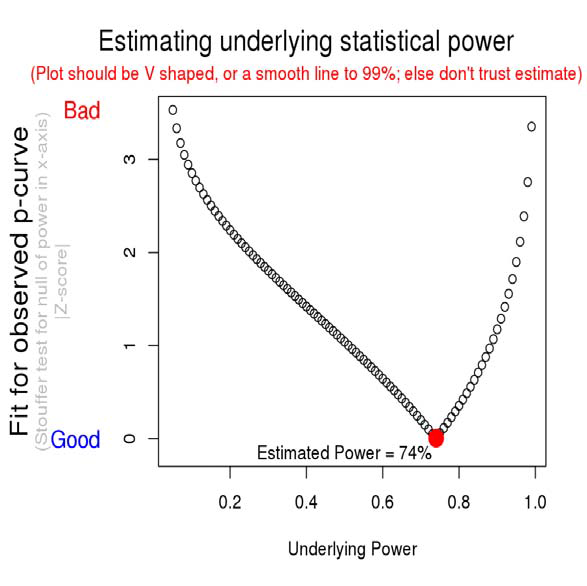


Notes: The figure above represents the estimated power for *p*-curve in which approximately 74% of the results (5 of 6 studies) have *p*-values <0.025. This suggests a lack of *p-*hacking.

Supplementary file 4. Plots for highest and lowest *p*-values.


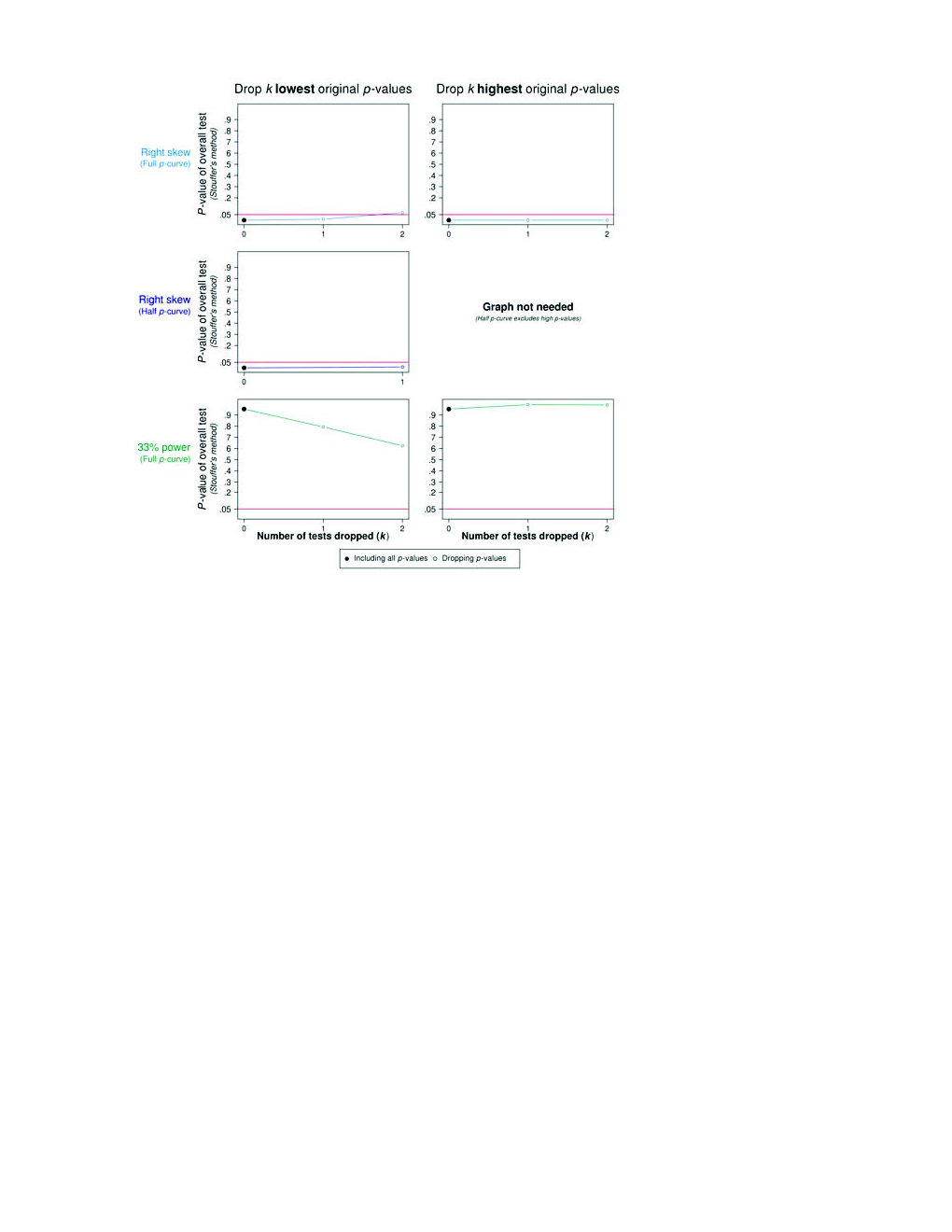


Notes: The figure panels above represent the sensitivity of findings by dropping the lowest and highest *p*-values from the analysis. The red, horizontal lines for all figures represent a *p*-value of 0.05. In the top two panels (full *p*-curve tests, blue line), a lack of *p-*hacking (<0.05) remains when dropping one, but not two, of the studies with the lowest *p*-values, while a lack of *p-*hacking remains when dropping the two studies with the highest *p*-values. For the middle panel (half *p*-curve test, purple line), a lack of *p-*hacking remains when dropping the one study with the lowest *p-*value from the analysis. For the bottom left panel (full *p*-curve test at 33% power, green line), the power for determining whether or not evidential value exists for *p*-hacking remains above 0.80 when dropping the one study with the lowest *p-*value from the analysis but drops below 0.70 when dropping the two studies with the lowest *p*-values from the analysis. For the bottom right panel, the power for determining whether or not evidential value exists for *p*-hacking remains above 0.90 when dropping the two studies with the highest *p*-values from the analysis.
